# Referred pain from myofascial trigger points in head and neck–shoulder muscles reproduces head pain features in children with chronic tension type headache

**DOI:** 10.1007/s10194-011-0316-6

**Published:** 2011-02-27

**Authors:** César Fernández-de-las-Peñas, Daniel M. Fernández-Mayoralas, Ricardo Ortega-Santiago, Silvia Ambite-Quesada, Domingo Palacios-Ceña, Juan A. Pareja

**Affiliations:** 1 Department of Physical Therapy, Occupational Therapy, Rehabilitation and Physical Medicine, Facultad de Ciencias de la Salud, Universidad Rey Juan Carlos, Avenida de Atenas s/n, 28922 Alcorcón, Madrid Spain; 2 Esthesiology Laboratory of Universidad Rey Juan Carlos, Alcorcón, Spain; 3 Department of Health Science and Technology, Centre for Sensory-Motor Interaction (SMI), Aalborg University, Aalborg, Denmark; 4 Hospital Quirón de Madrid, Madrid, Spain; 5 Centro “CADE”, Madrid, Spain; 6 Department of Anatomy, Universidad Rey Juan Carlos, Madrid, Spain; 7 Department of Health Sciences II, Universidad Rey Juan Carlos, Madrid, Spain; 8 Neurology Department, Hospital Fundación Alcorcón, Madrid, Spain

**Keywords:** Chronic tension-type headache, Trigger points, Referred pain, Children

## Abstract

Our aim was to describe the referred pain pattern and areas from trigger points (TrPs) in head, neck, and shoulder muscles in children with chronic tension type headache (CTTH). Fifty children (14 boys, 36 girls, mean age: 8 ± 2) with CTTH and 50 age- and sex- matched children participated. Bilateral temporalis, masseter, superior oblique, upper trapezius, sternocleidomastoid, suboccipital, and levator scapula muscles were examined for TrPs by an assessor blinded to the children’s condition. TrPs were identified with palpation and considered active when local and referred pains reproduce headache pain attacks. The referred pain areas were drawn on anatomical maps, digitalized, and also measured. The total number of TrPs was significantly greater in children with CTTH as compared to healthy children (*P* < 0.001). Active TrPs were only present in children with CTTH (*P* < 0.001). Within children with CTTH, a significant positive association between the number of active TrPs and headache duration (*r*_s_ = 0.315; *P* = 0.026) was observed: the greater the number of active TrPs, the longer the duration of headache attack. Significant differences in referred pain areas between groups (*P* < 0.001) and muscles (*P* < 0.001) were found: the referred pain areas were larger in CTTH children (*P* < 0.001), and the referred pain area elicited by suboccipital TrPs was larger than the referred pain from the remaining TrPs (*P* < 0.001). Significant positive correlations between some headache clinical parameters and the size of the referred pain area were found. Our results showed that the local and referred pains elicited from active TrPs in head, neck and shoulder shared similar pain pattern as spontaneous CTTH in children, supporting a relevant role of active TrPs in CTTH in children.

## Introduction

Tension-type headache is the most common form of headache in both adults [1] and adolescents [2]. Different studies have reported an overall prevalence rate for tension type headache ranging from 5.5 to 26% in children between 6 and 12 years old [3–6]. In a recent epidemiological study in Germany, the 6-month prevalence of headaches was 53.2% among children from 7 to 14 years [7]. In fact, Lewis et al. [8] estimated that about 20% of the children with primary headache need medical therapy. Furthermore, as tension type headache is also problematic for children, this headache needs further study [9, 10].

Although there has been an increasing interest in the pathogenic mechanisms of tension type headache, the true patho-anatomical mechanisms remain inconclusive [11]. It seems clear that hyper-excitability of peripheral and central nociceptive pain pathways plays an important role in tension type headache [12], as several studies have demonstrated the presence of pressure pain hyperalgesia in children with tension type headache [13–15].

Nevertheless, it has been postulated that tension type headache-related pain may be originated, at some extent, from referred pain from muscle trigger points (TrPs) located in head, neck and shoulder muscles [16, 17]. Myofascial/muscle TrPs are usually defined as the hypersensitive spots in a taut band of a skeletal muscle that elicit a referred distant pain upon examination [18]. From a clinical point of view, TrPs may be active or latent. Active TrPs are those which both local and referred pain reproduce pain symptoms and the pain is recognized as a usual or familiar pain by the subjects. In tension type headache, active TrPs are those reproducing pain symptoms similar to those the patients perceive during their headache attacks.

Different studies have demonstrated the relevance of active TrPs in adults with chronic tension type headache (CTTH) [19]. In fact, a series of studies reported that the referred pain elicited by active TrPs in suboccipital [20], upper trapezius [21], temporalis [22], superior oblique [23], and sternocleidomastoid [24] muscles reproduced the head pain pattern in CTTH. Although muscle TrPs may be also involved in the development of CTTH in children, the literature on this topic is scarce. In fact, a recent case series has suggested that myofascial TrPs may play an additional role in a subgroup of children with tension-type headache [25]. This study showed that treatment targeted at inactivating TrPs may be effective for reducing the intensity, duration, and frequency of headache in children with tension type headache. However, this was a non-controlled study which only included nine girls [25]. To the best of the authors’ knowledge; no previous study has investigated the referred pain areas of myofascial TrPs in children with CTTH in a systematically way. Our aims were to examine the presence of myofascial TrPs in head, neck, and shoulder muscles in children with CTTH and healthy controls and to compare the referred pain patterns and size of the areas in relation to clinical features of CTTH pain.

## Materials and methods

### Subjects

Consecutive children diagnosed with CTTH by an experienced paediatric neurologist from the Pediatric Neurology Department of the General Hospital Quirón were screened for eligibility criteria. In all children headache features, temporal profile of the headache, and family history were assessed. To be included children had to describe all the characteristics typical of CTTH according to the ICHD-II criteria [26]: bilateral location, pressing or tightening pain, mild/moderate intensity (≤6 on a numerical pain rate scale) and no aggravation of headache during physical activity. Only one, either photophobia or phonophobia, was permitted. No children reported vomiting or evident nausea during pain attacks. Other primary headaches, medication-overuse, and secondary headaches were excluded [26]. None of the children was taken prophylactic drugs at the time of the study.

Additionally, age- and sex- matched children without history of head or neck pain symptoms were recruited from volunteers who responded to a local announcement. Ethical Approval was granted by Local Ethics Committee (FHA 043). Informed consent was obtained from both the children and parents and all procedures were conducted according to the Declaration of Helsinki.

### Self-reported measures

Children completed a headache diary for 4 weeks in order to complement the diagnosis [27]. A 11-point numerical pain rate scale [28] (NPRS; range: 0 = no pain to 10 = maximum pain) was used to assess headache intensity. The diary was completed daily, irrespective of presence or absence headache with the assistance of their parents. It was recommended that the children fill out the diary once per day before going to bed to record the information for the whole day. One assessor made weekly telephone calls to increase compliance with filling out the daily diary.

The headache diary was used to calculate the following variables: (1) headache intensity, calculated from the mean of the NPRS of the days with headache; (2) headache frequency, calculated by dividing the number of days with headache by the number of the analyzed weeks (days/week) and (3) headache duration, calculated by dividing the sum of the total hours of headache by the number of days with headache (hours/day).

### Muscle trigger point examination

Muscle TrPs were bilaterally explored within the upper trapezius, temporalis, masseter, and sternocleidomastoid muscles by an examiner with more than 10 years experience in TrP examination, and who was blinded to the children’s condition. TrP diagnosis in these muscles was done following the criteria as described by Simons et al. [18] and by Gerwin et al. [29]: (1) presence of a palpable taut band in a skeletal muscle; (2) presence of a hyperirritable tender spot within the taut band; (3) local twitch response elicited by the snapping palpation of the taut band; and (4) presence of referred pain in response to TrP compression (approximately 20 N force for 5 s).

Additionally, TrPs within superior oblique and suboccipital muscles were also examined following previous guidelines [20, 23]. Briefly, TrP diagnosis in the superior oblique muscles was made when there was local tenderness in the trochlear region, referred pain with maintained pressure for 10 s and increased referred pain with both contraction (downward-medial gaze) and stretching (upward-lateral gaze) of the superior oblique muscle [23].

TrP diagnosis within the suboccipital muscles was made when there was local tenderness in the suboccipital region, referred pain with maintained pressure for 10 s and increased referred pain with active extension of the upper cervical spine [20].

TrPs were considered active if both the local and the referred pain evoked by the compression reproduced the spontaneous pain symptoms of the children and the elicited pain was recognized by the children [18], whereas TrPs were considered latent if the local and referred pain elicited by the compression did not reproduce any pain symptom familiar to the children [18].

TrP examination was performed in a blinded fashion. After TrP assessment on each muscle, children were asked by a second assessor: “When I pressed these muscles, did you feel any pain or discomfort locally, and in other area (referred pain). Please tell me whether the pain that you felt in the other area reproduced any symptoms that you suffer from.” Children had to indicate whether the pain elicited by palpation reproduced a familiar or usual pain or another non-familiar type of pain. In such a way, assessment of TrPs was performed in blinded fashion. The order of TrP evaluation was randomized between participants.

### Assessment of referred pain area and quality

Local pain was defined as a pain located around the compression site, and referred pain was defined as the pain located at least 1 cm outside the local pain area evoked by TrP palpation. Finally, children were asked to draw the distribution of the referred pain on an anatomical map after palpation of each TrP. The spontaneous pain symptoms and the referred pain areas were measured with a digitizer (ACECAD D9000, Taiwan) [30, 31].

### Statistical analysis

Data were analysed with the SPSS statistical package (16.0 Version). Results are expressed as mean, standard deviation (SD) or 95% confidence interval (95% CI). The Kolmogorov–Smirnov test was used to analyse the normal distribution of the variables (*P* > 0.05). Quantitative data without a normal distribution (i.e., pain history, headache intensity, headache frequency, headache duration, and number of active muscle TrPs) were analysed with non-parametric tests, and data with a normal distribution (referred pain areas) were analysed with parametric tests. Differences in the number of active TrPs between groups were assessed with the non-parametric Mann–Whitney *U* test. The Chi-square (χ^2^) test was used to assess the differences in the size of distribution of TrPs for each muscle on either side within both study groups. A 3-way analysis of variance (ANOVA) was used to compare the areas of referred pain (arbitrary units) between sides (dominant/non dominant) and muscles (i.e., temporalis, masseter, superior oblique, upper trapezius, and sternocleidomastoid) as within-subject factors and group (patients, controls) as between-subject factor. A similar 2-way ANOVA was used for the referred pain areas from the suboccipital muscles but without side as factor. The Bonferroni test was used for post-hoc analyses. The Spearman’s rho (*r*_s_) test was used to analyse the association between the number of TrPs, the referred pain areas and clinical variables of the headache. The statistical analysis was conducted at 95% confidence level. A *P* value less than 0.05 was considered statistically significant.

## Results

### Demographic and clinical data of the sample

Seventy (*n* = 70) consecutive children presenting with headache between May 2009 and March 2010 were screened. Twenty (28%) were excluded: migraine (*n* = 10), hemi-cranial headache (*n* = 3), depression (*n* = 3) or anxiety (*n* = 4). Finally, a total of 50 children, 14 boys and 36 girls, aged 6–12 years old (mean: 8 ± 2 years) satisfied all the inclusion criteria and agreed to participate. In our sample, headache history was 1.9 years (95% CI 1.7–2.2 years), the mean intensity per episode was 5.0 (95% CI 4.6–5.3), mean headache period per day was 4.2 h (95% CI 3.5–4.9 h), and the number of days per week with headache was 4.4 (95% CI 4.1–4.6 days/week). No significant associations between headache intensity, frequency or duration (*P* > 0.472) were found. The mean BDI-II score was 3.6 (95% CI 3.2–4.0). No significant associations between headache parameters and BDI-II were found (*P* > 0.381).

**Fig. 1:**
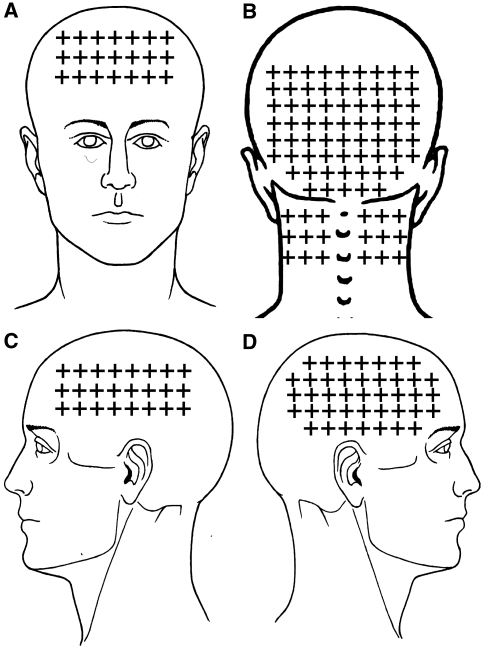
Areas showing the spontaneous pain symptoms of children with chronic tension type headache: **a** Frontal region; **b** Occipital region; **c** Non-dominant side; **d** Dominant side

The mean spontaneous pain area reported by CTTH children was 57.7 arbitrary units (95% CI 50.0–65.6) in the frontal region (*n* = 41, 82%), 59.8 (95% CI 44.8–74.8) in the occipital region (*n* = 24, 58%) including the posterior part of the neck), 32.4 (95% CI 25.8–39.1) on the dominant side of the head (*n* = 35, 70%), and 28.7 (95% CI 23.0–34.3) in the non dominant of the face (*n* = 33, 66%). The overall spontaneous pain area of children with CTTH is illustrated in Fig. 1. Significant positive correlations between headache intensity and spontaneous pain areas within dominant (*r*_s_ = 0.543; *P* < 0.001) and non dominant (*r*_s_ = 0.467; *P* = 0.005) sides were found: the higher the headache pain intensity, the larger the spontaneous pain area in both sides.

In addition, 50 age- and sex- matched children without headache, 14 boys and 36 girls, aged 6–12 years (mean: 8 ± 1.6 years old) also participated (*P* = 0.897).

### Muscle TrPs

The mean ± SD number of TrPs of each child with CTTH was 4 ± 2, being all of them active TrPs. Healthy children showed latent, but not active, TrPs (mean ± SD: 0.3 ± 0.7). Therefore, the total number of TrPs was significantly higher within children with CTTH (*z* = −8.939; *P* < 0.001). In children with CTTH, a significant positive association between the number of active TrPs and headache duration (*r*_s_ = 0.315; *P* = 0.026) was observed: the greater the number of active TrPs, the longer the duration of headache attack. No other association between the number of active TrPs and length of headache (*r*_s_ = 0.090; *P* = 0.533), headache intensity (*r*_s_ = 0.261; *P* = 0.067) or headache frequency (*r*_s_ = 0.220; *P* = 0.125) was found.

In all the examined muscles, active TrPs were only present within children with CTTH compared to healthy children (*P* < 0.001). The distribution of myofascial TrPs between patients and controls was significantly different for the upper trapezius (both sides: χ^2^ = 14.967, *P* < 0.001), sternocleidomastoid (dominant side: χ^2^ = 15.674, *P* < 0.001; non-dominant side: χ^2^ = 6.383, *P* = 0.012); temporalis (dominant: χ^2^ = 61.763, *P* < 0.001; non-dominant: χ^2^ = 51.515, *P* < 0.001), superficial masseter (dominant side: χ^2^ = 9.538, *P* = 0.008; non-dominant side: χ^2^ = 5.263, *P* = 0.022), suboccipital (χ^2^ = 66.780, *P* < 0.001), superior oblique (dominant: χ^2^ = 9.337, *P* = 0.009; non-dominant: χ^2^ = 10.494, *P* = 0.005), and dominant levator scapulae (χ^2^ = 6.383, *P* = 0.012) muscles, but not for the non-dominant levator scapulae muscle (χ^2^ = 0.709, *P* = 0.400). In fact, suboccipital muscle TrPs were the most prevalent (80%, *n* = 40), followed by temporalis TrPs (70%, *n* = 38 dominant side; 32%, *n* = 16 non-dominant side) and superior oblique muscle TrPs (28%, *n* = 14 dominant side; 30%, *n* = 15 non-dominant side) within children with CTTH. Table 1 summarizes the distribution of TrPs in both children with CTTH and healthy children.

**Table 1:** Number of children with chronic tension type headache and healthy children (*n*) with muscle trigger points (trps) located in head and neck-shoulder musculature

	Children with chronic tension type headache (CTTH)					
	Upper trapezius muscle		Sternocleidomastoid muscle		Temporalis	
	Dominant side	Non dominant side	Dominant side	Non dominant side	Dominant side	Non dominant side
Active TrPs (*n*)	10	10	13	6	38	16
No TrPs (*n*)	40	40	37	44	12	34

	Superficial masseter muscle		Superior oblique muscle		Levator scapulae muscle	
	Dominant side	Non dominant side	Dominant side	Non dominant side	Dominant side	Non dominant side
Active TrPs (*n*)	8	5	14	15	6	4
No TrPs (*n*)	42	45	36	35	44	46

	Healthy control children					
	Upper trapezius muscle		Sternocleidomastoid muscle		Temporalis	
	Dominant side	Non dominant side	Dominant side	Non dominant side	Dominant side	Non dominant side
Latent TrPs (*n*)	0	0	1	0	3	0
No TrPs (*n*)	50	50	49	50	47	50

	Superficial masseter muscle		Superior oblique muscle		Levator scapulae muscle	
	Dominant side	Non dominant side	Dominant side	Non dominant side	Dominant side	Non dominant side
Latent TrPs (*n*)	1	0	4	4	0	2
No TrPs (*n*)	49	50	46	46	50	48

### Referred pain area

**Fig. 2:**
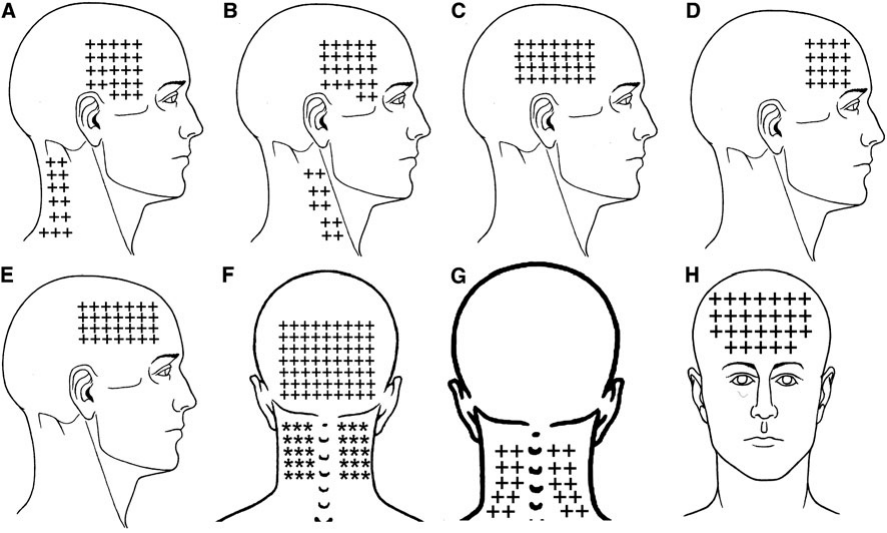
Referred pain from active TrPs in upper trapezius (**a**), sternocleidomastoid (**b**), temporalis (**c**), superficial masseter (**d**), suboccipital (**e**), combined suboccipital-upper trapezius (**f**), levator scapulae (**g**), and superior oblique (**h**) muscles in children with chronic tension type headache. *Note that the posterior referred pain in figure **f** comes from the suboccipital muscles (+) and from the upper trapezius (*)

The referred pain elicited by upper trapezius TrPs spread to the lateral aspect of the neck (10/10 both sides) and to the temple (8/10 both sides). Sternocleidomastoid muscle TrPs were associated with the referred pain to the temple (13/13 dominant side and 6/6 non-dominant side). Temporalis muscle TrPs referred pain to the temple perceived inside the head (38/38 dominant side, 16/16 non-dominant). The referred pain elicited by superficial masseter TrPs was perceived into the forehead (8/5 dominant side, 5/5 non-dominant side). Superior oblique muscle TrPs referred pain to the forehead (14/14 dominant side, 15/15 non-dominant side) and behind the eye (10, in both sides). Levator scapulae TrPs referred pain to the posterior part of the neck (6/6 dominant side, 4/4 non-dominant side). Finally, suboccipital muscle TrP referred pain was perceived inside the head particularly into the forehead and behind the eyes (40/40 bilaterally). Figure 2 depicts the referred pain patterns elicited by active TrPs. In fact, the combination of the referred pain patterns from active TrPs fully reproduced the overall spontaneous clinical pain pattern in children with CTTH (Fig. 1).

A 3-way ANOVA showed significant differences in referred pain areas between groups (*F* = 18.687, *P* < 0.001) and muscles (*F* = 3.239, *P* = 0.008), but not between sides (*F* = 0.264; *P* = 0.613). No significant interactions between group × muscle (*F* = 0.382, *P* = 0.821), group × side (*F* = 1.073, *P* = 0.317), side × muscle (*F* = 0.209, *P* = 0.958) or group × muscle × side (*F* = 0.064, *P* = 0.802) were found. A 2-way ANOVA for the suboccipital musculature revealed similar results (group: *F* = 36.551, *P* < 0.001; muscle: *F* = 4.211, *P* = 0.001; group × muscle: *F* = 1.735, *P* = 0.406). Thus, children with CTTH had larger pain areas compared to healthy children (*P* < 0.001). Bonferroni post-hoc analyses revealed that the referred pain area elicited by suboccipital muscle TrPs was significantly larger than the referred pain elicited from all other muscles (*P* < 0.001), except from levator scapulae muscle TrPs (*P* = 0.212). In addition, referred pain areas from the upper trapezius, sternocleidomastoid, temporalis, masseter, and superior oblique muscle TrPs were not significantly different in size (*P* > 0.312). Table 2 details the size of the referred pain areas in all the examined muscles.

**Table 2:** Referred pain areas of myofascial trigger points in head and neck–shoulder muscles in children with chronic tension type headache and healthy control children (*n* = 50)

	Children with chronic tension type headache^#^		Healthy controls children	
Upper trapezius	Dominant side (*n* = 10)	32.5 ± 15.8 (21.8–39.2)	Dominant side (*n* = 0)	–
	Non dominant side (*n* = 10)	28.8 ± 18.1 (18.6–39.1)	Non dominant side (*n* = 0)	–
Sternocleidomastoid	Dominant side (*n* = 13)	27.1 ± 14.6 (16.1–38.2)	Dominant side (*n* = 1)	12.2
	Non dominant side (*n* = 6)	29.1 ± 14.9 (19.7–38.5)	Non dominant side (*n* = 0)	–
Temporalis	Dominant side (*n* = 38)	32.6 ± 16.9 (26.7–38.6)	Dominant side (*n* = 3)	14.6 ± 8.1 (10.1–19.3)
	Non dominant side (*n* = 16)	31.1 ± 18.1 (25.4–36.9)	Non dominant side (*n* = 0)	–
Superficial masseter	Dominant side (*n* = 8)	22.9 ± 9.3 (14.4–31.5)	Dominant side (*n* = 1)	3.4
	Non dominant side (*n* = 5)	28.7 ± 12.9 (20.6–36.8)	Non dominant side (*n* = 0)	–
Superior oblique	Dominant side (*n* = 14)	38.2 ± 18.8 (26.9–49.4)	Dominant side (*n* = 4)	25.4 ± 9.1 (15.2–35.6)
	Non dominant side (*n* = 15)	35.5 ± 14.4 (24.7–46.3)	Non dominant side (*n* = 4)	26.6 ± 8.9 (18.5–34.7)
Levator scapulae	Dominant side (*n* = 6)	52.0 ± 11.5 (32.4–71.6)	Dominant side (*n* = 0)	–
	Non dominant side (*n* = 4)	55.1 ± 16.6 (29.7–70.4)	Non dominant side (*n* = 2)	22.8 ± 6.1 (11.1–36.7)
Suboccipital*	Bilateral (*n* = 10)	75.4 ± 18.3 (64.2–86.6)	Bilateral (*n* = 1)	16.2

Referred pain areas (arbitrary units) are expressed as means ± standard deviation (95% confidence interval)

^#^Significant differences between groups (*P* < 0.001)

* Significant differences with the remaining muscles (*P* < 0.001)

Finally, significant positive correlations between headache clinical parameters and the size of the referred pain area were found: (a) years with headache were significantly associated with the referred pain area in both upper trapezius muscles (*r*_s_ = 0.700; *P* = 0.011); (b) headache intensity was significantly associated with the size of the referred pain area in both sternocleidomastoid muscles (*r*_s_ = 0.753; *P* = 0.007); (c) the duration of headache was significantly associated with the referred pain area from both masseter (*r*_s_ = 0.763; *P* = 0.017) and superior oblique (*r*_s_ = 0.599; *P* = 0.024) muscles. In summary, the greater the length of the onset of headache, the higher headache intensity, or the longer the mean duration of headache, the larger the referred pain areas elicited by TrP palpation.

## Discussion

The current controlled and blinded study showed the existence of multiple active TrPs in head and neck-shoulder muscles in children with CTTH. Both local and referred pain characteristics elicited from manual palpation of active TrPs reproduced the pain pattern in all children with CTTH. Additionally, TrP referred pain areas were larger in children with CTTH than in healthy children. The size of referred pain areas of some muscles was positively related to some headache clinical parameters.

A recent study has found that children with CTTH exhibit lesser cervical range of motion than children without headache, particularly in flexion/extension and lateral-flexion [32]. It may be possible that the presence of TrPs in the neck muscles can reduce cervical range of motion [18]. In the current study, we found active TrPs in the upper trapezius and sternocleidomastoid muscles in children with CTTH, which may reduce extension and lateral-flexion motions. Future studies should investigate the relationship between restricted cervical range of motion and the presence of active TrPs in neck and shoulder muscles in children with headache.

### Referred pain from active TrPs in children with CTTH

Active myofascial TrPs in head, neck and shoulder muscles elicited a referred pain that reproduced headache pain pattern in children with CTTH. When active TrPs were explored, children reported: “Yes, this is the pain that I feel during pain attacks.” These findings support the view that active TrPs in these muscles may be involved in the pathophysiology and manifestation of CTTH pain in children. In fact, an important finding was that children were explored headache-free, which increases the relevance of active TrPs in the development of CTTH. In addition, our results are further supported by a previous pilot study where treatment targeted at inactivating TrPs was effective for reducing the intensity, duration and frequency of headache in nine girls with tension type headache [25].

Active muscle TrPs were not found in healthy children, since they did not suffer from any pain symptoms. Nevertheless, latent TrPs were observed in a few subjects in some of the explored muscles, particularly the superior oblique muscle. It has been proposed that latent TrPs may become active under the influence of several factors such as muscle overload or strain. Therefore, the presence of latent TrPs in healthy children may be potentially implicated in posterior development of pain symptom, although longitudinal studies are needed to confirm this hypothesis.

In the current study, TrPs in the suboccipital, temporalis and superior oblique muscles were the most prevalent in our sample of children with CTTH. These results are very similar to those previously found in adults with CTTH where suboccipital [20] and temporalis [22] muscles were also the most prevalent. Nevertheless, children with CTTH showed less percentage of active TrPs in the upper trapezius muscle as compared to adults with CTTH [21].

We also found that referred pain areas from suboccipital TrPs were larger than the pain areas from the remaining muscle TrPs and that those children with CTTH showed larger areas of referred pain as compared to healthy children. Current findings support that sensitization mechanisms are involved in pain spreading or pain referral in children with CTTH.

### Peripheral and central sensitization associated with muscle TrPs in CTTH

The results of the current study likely reflect the presence of both peripheral and central sensitization mechanisms in children with CTTH. The presence of active TrPs indicate sensitization of peripheral muscular nociceptors since high levels of chemical mediators [33, 34] and greater mechanical hypersensitivity [35, 36] have been found in active TrPs. Additionally, a study has recently demonstrated the existence of both nociceptive (hyperalgesia) and non-nociceptive (allodynia) hypersensitivity at TrPs [37]. These studies support that active TrPs constitute a focus of peripheral sensitization of both nociceptive and non-nociceptive nerve endings, which may constitute a peripheral nociceptive drive into the trigeminal nucleus caudalis.

In addition, referred pain phenomena from active TrPs may be also related to the presence of central sensitization. Graven-Nielsen et al. [38] found that the area of the referred pain is associated with the intensity of local pain, although this could not be shown for all the examined muscles in the present study. Discrepancies between experimental pain models and clinical studies including chronic pain patients are usually reported in the literature. Further, larger referred pain areas are also considered manifestation of central sensitization mechanisms [39]. In the current study, we showed that children with CTTH pain showed larger muscle referred pain areas in head, neck and shoulder muscle TrP as compared to healthy children, supporting a plausible role of central sensitization in muscle referred pain. In fact, current results agree with previous findings in adults with CTTH, where larger referred pain areas elicited from TrPs in the upper trapezius [21] and temporalis [22] muscles were also found. It is possible that the central sensitization is involved in larger referred pain areas elicited by active TrPs in CTTH. Interestingly, a brain imaging study found a distinct somatotopic organization of muscle referred pain areas as compared to local pain areas supporting the relevance of cortical processing in referred pain phenomenon [40]. Therefore, and not in contrast to peripheral sensitization process, central sensitization may also be involved in the generation of TrP referred pain in children with CTTH.

We found up to four active muscle TrPs within each children with CTTH which supports the assumption of spatial summation of TrP activity in CTTH. Since active TrPs may be considered a peripheral nociceptive input [33, 34], the presence of multiple active TrPs may exert a spatial summation of nociceptive barrage to the trigemino-cervical nucleus caudalis. In fact, Fernández-de-las-Peñas et al. [17] formulated a pain model for CTTH involving peripheral sensitization from active TrPs and central sensitization mechanisms. According to their model, active TrPs in those muscles innervated by C1–C3 segments or the trigeminal nerve would be responsible for peripheral nociception creating a continuous and prolonged nociceptive afferent barrage into the trigemino-cervical nucleus caudalis, which will sensitize the central nervous system [17]. It is possible that similar sensitization processes also occur in children with CTTH, although longitudinal studies are needed to further elucidate the role of active TrPs in children with CTTH.

We must recognize some potential limitations of this study. First, only children with CTTH were included. It would be interesting to investigate the presence of referred pain elicited by active TrPs in children with frequent episodic tension type headache or migraine. In addition, we cannot establish a cause-and-effect relationship between TrPs and CTTH, because the design of the study was not longitudinal. Future controlled clinical studies should analyze the effects of TrP treatment in CTTH to further elucidate the etiologic role of active TrPs in this patient population.

## Conclusion

The current controlled and blinded study showed the existence of multiple active TrPs in head, neck and shoulder musculature in children with CTTH. Both local and referred pain characteristics elicited by palpation of active muscle TrPs reproduced the head pain patterns in children with CTTH. Referred pain areas elicited by active TrPs were larger in children with CTTH as compared to healthy children. The size of referred pain areas of some muscles was positively related to some headache clinical parameters. Our results support a role of active TrP in children with CTTH.
